# Prevention and Treatment of Insanity of Pregnancy and Puerperal Period

**Published:** 1897-05

**Authors:** 


### PREVENTION AND TREATMENT OF INSANITY OF PREGNANCY AND PUERPERAL PERIOD.

Dr. George H. Savage, in *British Gynecological Journal*, 1896, gives the following classification as embraced under the above heading thus dividing the mental disorders.

1. Occurring in the early or latter months of pregnancy.
2. Insanity of labor.
3. As associated with febrile disorders, as the establishment of milk secretion.
4. Appearing within the first two weeks previous to labor.
5. Insanity of lactation and that of weaning.

The writer questions the existence of a special definite form of insanity deserving the name of "puerperal mania." With reference to the predisposing causes, they are regarded similar for all the forms of the disturbance. Heredity is given as the most important factor.

First pregnancies, if occurring after the age of thirty, are considered more dangerous than those of an earlier age.

The induction of premature labor as a cure for insanity is not regarded as justifiable in every case.

Within the first two weeks following delivery, maniacal symptoms are most common with puerperal insanity; if developed later, melancholia is more frequent.

The writer questions the advisability of the marriage of neurotics, especially if there be a pronounced hysterical tendency.

The terms puerperal insanity may be ascribed more justly to a group having sepsis as the etiological factor.

The questions discussed as to the mode of treatment refers to etiology, the use of hypnotic drugs, the removal from home to more favorable surroundings, or the placing of patient in an asylum.—*Langsdale's Lancet.*

